# Risk assessment of biotechnology products

**DOI:** 10.1186/2045-7022-1-S1-S13

**Published:** 2011-08-12

**Authors:** Corinne Herouet-Guicheney

**Affiliations:** 1 Bayer S.A.S, Bayer CropScience, BioScience Regulatory Toxicology Bioscience, Sophia-Antipolis Cedex, France

## 

A number of crop products generated by using biotechnology have been introduced to the marketplace. These biotechnology products have been carefully evaluated for their overall safety from an agronomic, environmental, performance, and equivalence perspective, and the safety of the newly expressed protein(s). One aspect of the safety evaluation is the question concerning potential allergenicity. The allergy risk to consumers from crops enhanced through biotechnology may be placed into one of three categories. The first category involves the transfer of a known allergen or cross-reacting allergen into a food crop. The second risk category is the potential for increasing the allergenicity of a crop by increasing the expression of endogenous allergens. The last category involves expression of transgenic proteins that may become allergens de novo. Approaches to identifying potential food allergens for purposes of safety assessment have been developed and modified over the past 15 years. However, no single factor has been recognized as the primary indicator for the allergenic potential of proteins, and no validated animal model that is predictive of protein allergenicity is currently available. Therefore, the evaluation of protein allergenicity is currently based upon a ‘weight of evidence’ approach, which takes into account a variety of factors that have been associated with allergens, such as stability to pepsin digestion or other enzymes in vitro, glycosylation status, food processing effects, protein abundance in the crop, homology to known allergens, and the safety of the gene(s) source. In addition, as part of the ‘weight-of-evidence’ assessment, the biotechnology crop product endogenous allergen levels are compared to those of its non-biotechnology comparator to make sure that the transformation does not impact known endogenous allergens naturally present in the host crop. This presentation will provide a general introduction and overview of the current ‘state of the art’ for evaluating potential allergenicity of biotechnology crop products.

